# The effect of the flavonol rutin on serum and liver iron content in a genetic mouse model of iron overload

**DOI:** 10.1042/BSR20210720

**Published:** 2021-07-09

**Authors:** Zachary J. Hawula, Eriza S. Secondes, Daniel F. Wallace, Gautam Rishi, V. Nathan Subramaniam

**Affiliations:** Centre for Genomics, School of Biomedical Sciences, Faculty of Health, Queensland University of Technology (QUT), Brisbane, Queensland 4059, Australia

**Keywords:** flavonol, hereditary hemochromatosis, iron chelators, iron overload, rutin, transferrin receptor 2

## Abstract

The flavonol rutin has been shown to possess antioxidant and iron chelating properties *in vitro* and *in vivo*. These dual properties are beneficial as therapeutic options to reduce iron accumulation and the generation of reactive oxygen species (ROS) resultant from excess free iron. The effect of rutin on iron metabolism has been limited to studies performed in wildtype mice either injected or fed high-iron diets. The effect of rutin on iron overload caused by genetic dysregulation of iron homoeostasis has not yet been investigated. In the present study we examined the effect of rutin treatment on tissue iron loading in a genetic mouse model of iron overload, which mirrors the iron loading associated with Type 3 hereditary haemochromatosis patients who have a defect in Transferrin Receptor 2 (TFR2). Male TFR2 knockout (KO) mice were administered rutin via oral gavage for 21 continuous days. Following treatment, iron levels in serum, liver, duodenum and spleen were assessed. In addition, hepatic ferritin protein levels were determined by Western blotting, and expression of iron homoeostasis genes by quantitative real-time PCR. Rutin treatment resulted in a significant reduction in hepatic ferritin protein expression and serum transferrin saturation. In addition, trends towards decreased iron levels in the liver and serum, and increased serum unsaturated iron binding capacity were observed. This is the first study to explore the utility of rutin as a potential iron chelator and therapeutic in an animal model of genetic iron overload.

## Introduction

Iron is a fundamental micronutrient for all organisms; it is involved in several essential functions such as oxygen metabolism, electron transfer and in enzymes important for DNA and RNA synthesis [1]. Iron dysregulation can result in numerous clinical disorders including anaemia and haemochromatosis. Iron overload is one of the most common metal-related toxicities and is often caused by genetic defects in iron absorption, parenteral iron administration (typically resulting from transfusion-dependent anaemias) or pathological conditions characterised by increases in iron [2].

Flavonoids are naturally occurring polyphenolic phytochemicals found in fruits and vegetables as well as drinks including tea and red wine [3,4], where they provide colour and flavour to these foods [5]. The flavonoid family of compounds also have broad pharmacological activities and have been shown to be beneficial in numerous diseases including diabetes mellitus, allergy, cancer, viral infections, headache, stomach and duodenal ulcer, parodentosis and inflammation [4,5]. Pharmacological activities typical of flavonoids include interactions with enzymes, hormone carriers, DNA, antioxidant (free radical scavenging) and iron chelating properties [6–9]. The latter makes these compounds interesting options for the treatment of iron overload disorders.

Rutin, also known as rutoside, quercetin-3-*o*-rutinoside or sophorin is a flavonol glycoside [10]. It is commonly found in plants such as buckwheat and tobacco [11] and is also found in several herbal teas [4]. The five golden flowers tea (which contains approximately 1.55 mg/g dry weight rutin) has been found to display hepatoprotective properties including decreased aspartate transaminase levels [12]. Other edible flowers such as *Sambucus nigra* and *Hedysarum coronarium* which also contain high rutin content have been found to inhibit both α-amylase and α-glucoside expression [13]. In recent years, rutin has also become a potential therapeutic treatment option for various cancers due to its ability to target various apoptotic, autophagic and inflammatory markers including, nuclear factor-ϰB, tumour necrosis factor-α, light chain3/Beclin and various interleukins [9]. However, for this study rutin iron chelating properties were investigated. Several *in vitro* studies by us and others have been conducted which indicate that rutin possesses iron chelating activity [14–16]. Hussein et al. [17,18] have shown that orally administered rutin significantly decreased serum and liver iron, total iron binding capacity (TIBC), transferrin (Tf), transferrin saturation (TS) and ferritin protein levels in ferric hydroxide polymaltose-induced iron loaded male albino rats. In addition, Gao et al*.* found treatment with rutin significantly decreased hepatic iron levels in female Kunming mice [19]. Oral rutin treatment in male diabetic ApoE knockout mice significantly reduced total non-haem iron within the diabetic cohort [20]. However, these models do not accurately reflect acquisition of iron in genetic iron overload disorders. To our knowledge, no study has been conducted which investigates the role of flavonoids in the context of genetic iron overload. This presents an opportunity to better understand the therapeutic potential of rutin as genetic mutations that dysregulate iron homoeostasis are more likely to lead to iron overload in humans.

Transferrin receptor 2 (TFR2) is a homologue of ransferrin receptor 1 (TFR1), the primary cellular iron uptake protein [21]. TFR2 is expressed primarily in hepatocytes and erythroid precursor cells [22], where it has been suggested to play a role in the monitoring of iron through holo-Tf levels [23]. Our group has previously detailed the generation of a total *Tfr2* knockout (KO) mouse model, which displays no Tfr2 protein expression [24]. These mice have significant iron overload typical of humans with Type 3 hereditary haemochromatosis (HH) [24]. In this study, we examined the *Tfr2* KO mouse model of iron overload to determine whether oral administration of rutin can be used to rescue mice from iron overload typical of that seen in Type 3 HH patients.

## Materials and methods

### Experimental animals

*Tfr2*^−/−^ male mice on a C57BL6 strain background [24] (*n*=6 for each group) were housed and experiments performed at the University of Queensland Biological Research Facility (UQBRF) at the Translational Research Institute (TRI), Brisbane. Male mice were chosen for the present study as Hahn et al. have previously shown no gender differences in C57BL6 mice in the liver, brain, heart and retina [25]. All experimental procedures were approved by the QUT and UQ Animal Ethics Committee (approval number QUT/TRI/511/16). Animals received ethical, humane and responsible care according to the criteria outlined in the ‘Australian Code for the Care and Use of Animals for Scientific Purposes, 2013’. Animals had free access to standard laboratory water and food and were housed under a 12-h light/dark cycle. At 5 weeks of age, mice were either given a daily oral gavage of sterile water (vehicle control) or 60 mg/kg (body weight) rutin hydrate (Glentham Life Sciences, Wiltshire, United Kingdom) in a suspension of sterile water for 3 weeks (21 continuous days). The above-mentioned doses of rutin were selected on the basis of previous studies and reports which showed rutin reduces iron content within these animals [17,18,26,27]. Mice were weighed each day to determine quantity of rutin treatment, the daily oral gavage was performed by the technical staff at UQBRF. Underweight mice at the commencement of treatment were given wet mash (nutritional value identical to normal feed) instead of dry food to encourage weight gain. Rutin hydrate suspension was made fresh immediately before oral administration by adding sterile water and vortexing. After treatment (at 8 weeks of age), mice were killed, and blood and tissues collected for further analysis. The tissues were snap frozen in dry ice and stored at −80°C until time of analysis.

### Haematological parameters

Haematological parameters were measured using a Mindray BC-5000 Vet Haematology Analyser (Mindray Medical International Limited, Shenzhen, China) at TRI.

### Serum and tissue iron indexes

Serum iron and transferrin saturation was measured using an iron/total iron‐binding capacity reagent kit (Pointe Scientific, Canton, Michigan) as per the manufacturer’s instructions. Hepatic (HIC), duodenal (DIC), and splenic (SIC) iron concentrations were determined using the method of Torrance and Bothwell [28].

### Histological staining

Liver, duodenum, and spleen were fixed in formalin (10%), processed and sectioned at the Histological Facility at the QIMR Berghofer Medical Research Institute. Tissues were stained using Perls’ Prussian Blue for iron deposits as previously described by McDonald et al. [29]. Slides were counter-stained with nuclear fast red (Sigma–Aldrich, St. Louis, Missouri) for 5 min and mounted with Depex (Sigma–Aldrich). The sections were analysed by CaseViewer software (3DHisTech, Budapest, Hungary).

### Real-time PCR

Total RNA was isolated from liver using TRIzol (Life Technologies, Carlsbad, California) and isopropanol precipitation. cDNA (using 1 µg total RNA from liver) was synthesised using the SensiFAST cDNA synthesis kit (Bioline, Sydney, NSW, Australia) with real-time quantitative PCR (qPCR) performed using the SensiFAST SYBR No-Rox kit (Bioline). The expression levels of all target genes were calculated relative to the geometric mean of the three reference genes, β-actin (*Actb*), hypoxanthine-guanine phosphoribosyl transferase (*Hprt*) and DNA-directed RNA polymerase II subunit RPB1 (*Polr2a*) using the 2^−Δ*C*_t_^ method. Primers for detecting target genes are listed in Table 1.

**Table 1:** Real-time PCR primer sequences

Hamp-F	AGAGCTGCAGCCTTTGCAC
*Hamp*-R	ACACTGGGAATTGTTACAGCATTTA
*Bmp6*-F	ATGGCAGGACTGGATCATTG
*Bmp6*-R	CCATCACAGTAGTTGGCAGCG
*Tfr1*-F	CATGAGGGAAATCAATGATCGTA
*Tfr1*-R	GCCCCAGAAGATATGTCGGAA
*Tfr2*-F	CTATCTGGTCCTGATCACCCT
*Tfr2*-R	TCAGGGTTGACATCTTCATCGA
*Fpn1*-F	TTGCAGGAGTCATTGCTGCTA
*Fpn1*-R	TGGAGTTCTGCACACCATTGAT
*FtnH*-F	AAGTGCGCCAGAACTACCAC
*FtnH*-R	GCCACATCATCTCGGTCAA
*Smad7*-F	ACGGGAAGATCAACCCCGAG
*Smad7*-R	TTCCGCGGAGGAAGGTACAG
*Id1*-F	TCGTCGGTGGAACACATG
*Id1*-R	ACCCTGAACGGCGAGATCA
*Zip14*-F	GACAATTACGTCTCCAAGTCTGC
*Zip14*-R	CTTCTTGGAAGGAAGTGTCTCG
*Actb*-F	GACGGCCAAGTCATCACTATTG
*Actb*-R	CCACAGGATTCCATACCCAAGA
*Hprt*-F	GGACTGATTATGGACAGGA
*Hprt*-R	GAGGGCCACAATGTGATG
*Polr2a*-F	AGCTGGTCCTTCGAATCCGC
*Polr2a*-R	CTGATCTGCTCGATACCCTGC

### Western blotting

Liver homogenates (20 µg) were electrophoresed on 12% SDS/polyacrylamide gels. Proteins were then transferred on to nitrocellulose membranes (0.2 µm pore size) (Bio-Rad Laboratories, Gladesville, NSW, Australia) using a Trans-blot Turbo (Bio-Rad) blotting system. The membrane was then blocked with 10% skim milk in Tris buffered saline with 0.1% Tween-20 (TBST) (Sigma–Aldrich)) and then incubated with anti-GAPDH (1:360000, Merck Millipore, Bayswater, Victoria, Australia) and anti-ferritin (1:5000, Cell Signaling, Danvers, Massachusetts) diluted in 10% skim milk overnight at 4°C. The membrane was washed thrice with TBST before being incubated with secondary anti-rabbit or anti-mouse IgG conjugated to horseradish peroxidase (1:1000) (65-6120, Invitrogen, Waltham, Massachusetts) diluted in 10% skim milk for 1 h at room temperature. The membrane was further washed before being incubated with chemiluminescent substrate (Lumina Forte; Merck Millipore) and imaged on a Chemidoc imaging system (Bio-Rad) for various timepoints. The blots were quantitated using ImageJ (National Institutes of Health, Bethesda, Maryland).

### Statistical analyses

Statistical analysis on variables between treatment groups were analysed using an unpaired Student’s *t* test within GraphPad Prism 8.4.3 software (GraphPad Software, San Diego, CA) with *P*-values <0.05 considered statistically significant.

## Results

### Haematological parameters

**Figure 1:**
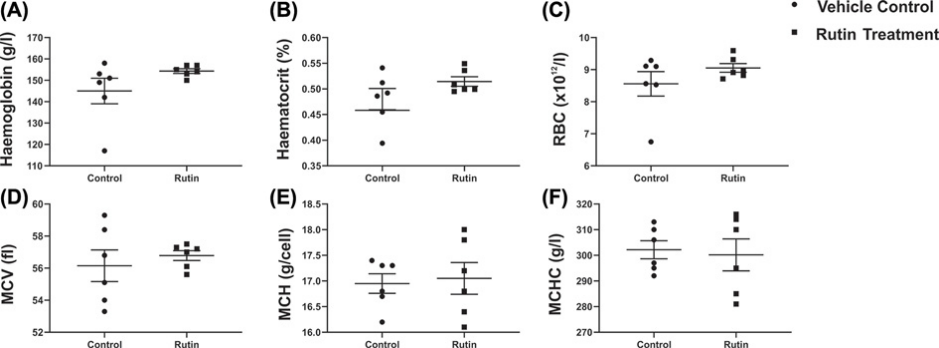
Haematological parameters of water- and rutin-treated *Tfr2* KO mice Effect of rutin treatment by oral gavage for 21 consecutive days on (**A**) haemoglobin, (**B**) haematocrit, (**C**) RBC (red blood cell) count, (**D**) MCV (mean cell volume), (**E**) MCH (mean cell haemoglobin) and (**F**) MCHC (mean cell haemoglobin concentration) were measured in *Tfr2* KO male mice treated with either rutin hydrate or vehicle control (*n*=6 in each group) by oral gavage for 21 consecutive days. Data are shown as dot plots, with lines indicating the mean and standard error of the mean (SEM). No statistically significant differences were observed between groups using an unpaired Student’s *t* test (*P>0.05*).

We first performed a haematological analysis on rutin and vehicle-treated mice. This analysis indicated that rutin treatment for 21 days did not significantly affect haematological parameters of the treated mice. As can be seen in Figure 1, haemoglobin levels, red blood cell (RBC) count, haematocrit, mean cell haemoglobin (MCH), mean cell volume (MCV) and mean cell haemoglobin concentration (MCHC) were all comparable between rutin- and vehicle-treated control mice. No changes were detected in neutrophil, monocyte and basophil levels after rutin treatment (data not shown).

### Assessment of iron status in the liver, spleen, duodenum and serum

**Figure 2:**
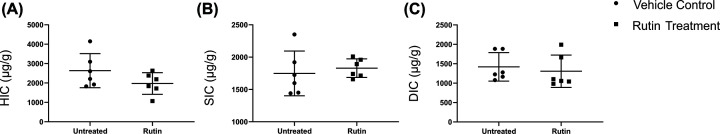
Tissue iron indices Effect of rutin treatment by oral gavage for 21 consecutive days on (**A**) HIC (hepatic iron concentration), (**B**) SIC (splenic iron concentration), and (**C**) DIC (duodenal iron concentration) were measured in *Tfr2* KO male mice treated with either rutin hydrate or vehicle control (*n*=6 in each group) by oral gavage for 21 consecutive days. Data are shown as dot plots, with lines indicating the mean and standard error of the mean. HIC displayed a trend towards decreased iron content using unpaired Student’s *t* test (*P=0.15*).

**Figure 3:**
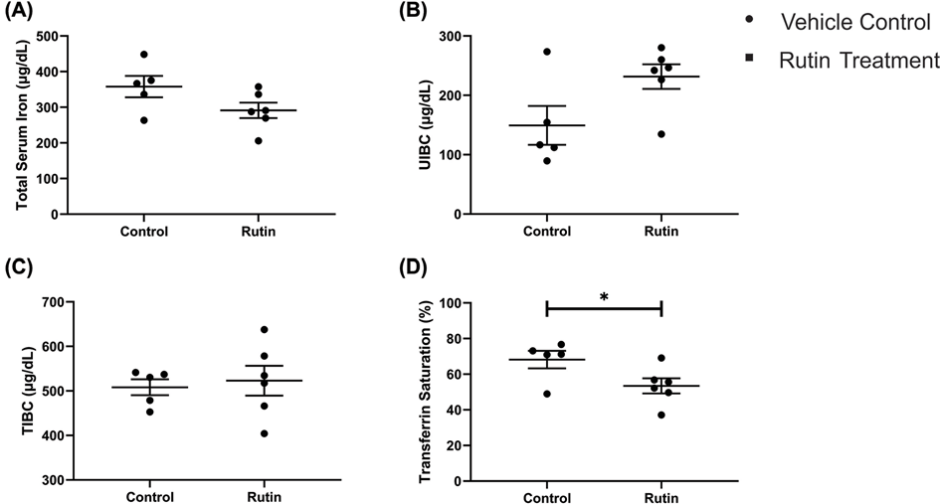
Serum iron indices Effect of rutin treatment by oral gavage for 21 consecutive days on (**A**) total iron serum content, (**B**) UIBC (unsaturated iron binding capacity), (**C**) TIBC (total iron binding capacity) and (**D**) transferrin saturation in *Tfr2* KO male mice (*n*=5 vehicle control; *n*=6 rutin treated). One control mouse was removed from analysis due to haemolysis of the blood sample. Data are shown as dot plots, with lines indicating the mean standard error of the mean. Statistically significant differences (unpaired Student’s *t* test (*P*<0.05)) are denoted with *.

We then determined the tissue iron concentration using colorimetric assays to detect total ferrous iron within the liver, spleen and duodenum (Figure 2). Total serum iron, total iron binding capacity (TIBC), unsaturated iron binding capacity (UIBC) and transferrin saturation (TS) were also measured (Figure 3). Rutin treatment resulted in a trend towards decreased HIC (2635.39 µg/g SEM ± 404.15 vs 1973.91 µg/g; SEM ± 307.41; *P*=0.15), while the SIC (1747.62 µg/g SEM ± 149.49 vs 1829.13 µg/g; SEM ± 79.58; *P*=0.60) and DIC (1419.65 µg/g SEM ± 143.19 vs 1307.00 µg/g; SEM ± 117.35; *P*=0.63) were similar in both vehicle- and rutin-treated mice.

Analysis of the serum iron levels (Figure 3) identified a trend towards decreased total serum iron (*P*=0.098) and increased UIBC (*P*=0.0554) within the rutin-treated group. Importantly the rutin-treated group displayed significantly decreased TS (*P*=0.0485) (Figure 3D). In the analysis of the serum iron indices, one mouse within the control group had to be removed due to haemolysis of the blood sample.

### Perls’ staining shows reduced liver iron after rutin treatment

**Figure 4:**
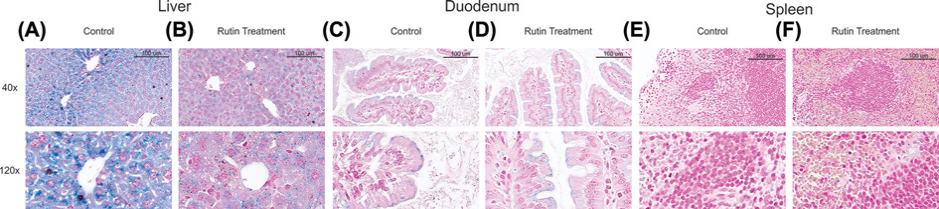
Histological staining for iron Perls’ staining of (**A**,**B**) liver, (**C**,**D**) duodenum and (**E**,**F**) spleen sections from representative *Tfr2* KO male mice treated with either vehicle control (A,C,E) or rutin hydrate (B,D,F) by oral gavage for 21 consecutive days, demonstrating decreased liver iron within the rutin-treated group as compared with the control group while no change can be seen in the spleen and duodenum. Scale bars = 100 µm.

The pattern of hepatic iron loading was assessed by Perls’ Prussian Blue staining of a representative liver section from each group (based on mean HIC) (Figure 4A,B). This indicated that there was decreased ferric iron within the rutin-treated group as compared with the control group (as denoted by reduction in blue staining pattern). Perls’ staining of the duodenum (Figure 4C,D) and spleens (Figure 4E,F) of these mice did not display altered ferric iron distribution between the control and rutin-treated groups.

### mRNA expression of iron homoeostasis-related genes is unaffected by rutin treatment

**Figure 5:**
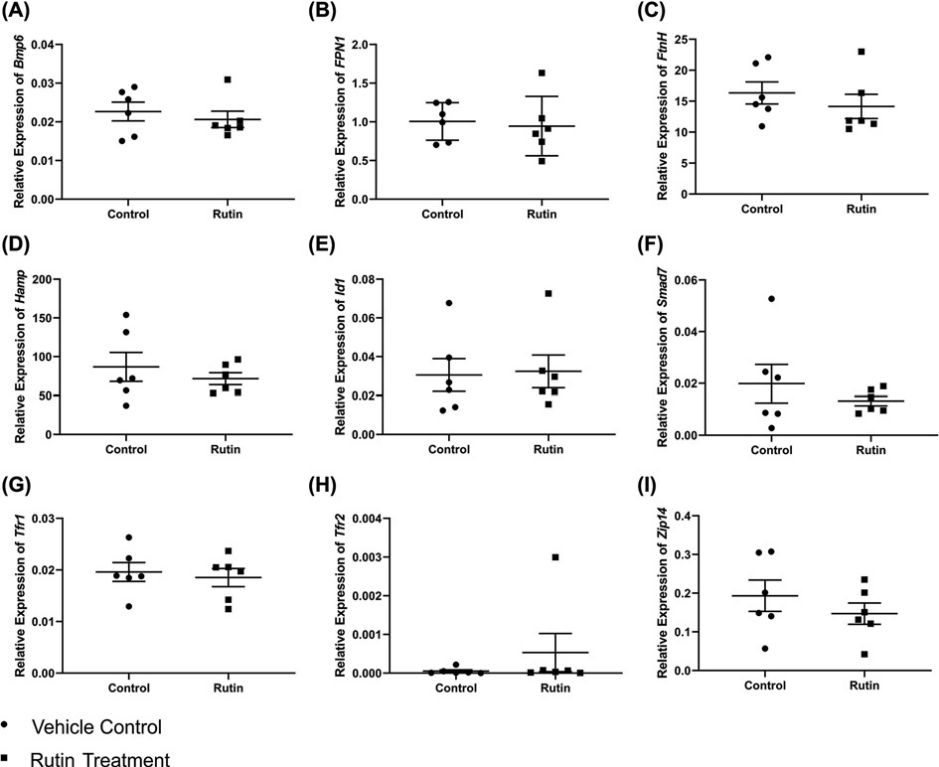
mRNA expression of iron homeostasis genes Expression of genes known to modulate iron homoeostasis within the livers of *Tfr2* KO male mice treated either rutin hydrate or vehicle control (*n*=6) by oral gavage for 21 consecutive days. mRNA expression levels of (**A**) *Bmp6*, (**B**) *Fpn1*, (**C**) *FtnH*, (**D**) hepcidin (*Hamp*), (**E**) *Id1*, (**F**) *Smad7*, (**G**) *Tfr1*, (**H**) *Tfr2* and (**I**) *Zip14* (relative to the geometric mean of three reference genes: *Actb, Hprt*, and *Polr2a*). Data are shown as dot plots, with line indicating the mean value and error bars indicating the standard error of the mean. No statistically significant differences were observed between groups using an unpaired Student’s *t* test (*P*>0.05).

The mRNA levels of genes known to regulate iron homoeostasis were then measured in the livers of rutin- and vehicle-treated *Tfr2*-KO mice using qPCR. No *Tfr2* mRNA was present in either control or rutin-treated mice (Figure 5H) indicating mice were true knockouts. As can be seen in Figure 5, bone morphogenetic protein 6 (*Bmp*6), ferroportin (*Fpn*), ferritin heavy chain (*FtnH*), inhibitor of DNA binding 1 (*Id1*), SMA and Mothers Against Decapentaplegic homologue 7 (*Smad7*), *Tfr1* and Zrt‐, Irt‐like protein‐14 (*Zip14*) mRNA levels did not differ significantly between the control and rutin-treated groups. *Hamp* expression was also not significantly changed after rutin treatment (Figure 5D).

### Liver ferritin expression decreases upon rutin treatment

**Figure 6:**
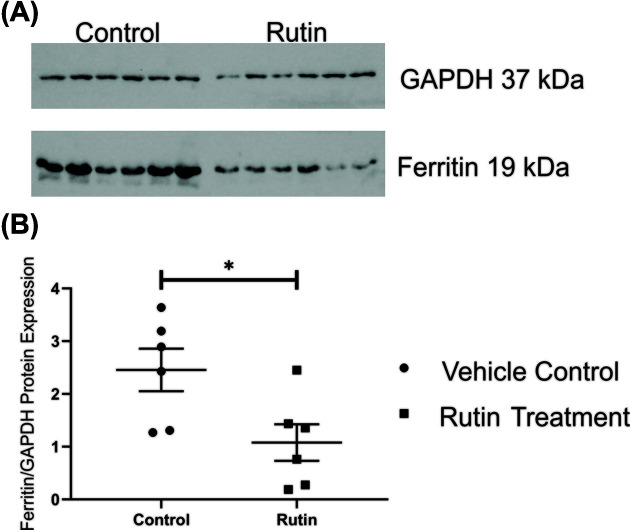
Western blot analysis of ferritin Protein expression of ferritin within the livers of *Tfr2* KO male mice treated with either rutin hydrate or vehicle control (*n*=6) by oral gavage for 21 consecutive days. (**A**) Western blot analysis of 20 µg of mouse liver homogenates with antibodies against ferritin and GAPDH as a loading control, (**B**) quantification of protein levels after normalising to GAPDH. Rutin treatment resulted in significantly reduced ferritin protein levels in treated group as compared with the control group. Data are shown as dot plots, with lines indicating the mean and standard error of the mean. Statistically significant differences (unpaired Student’s *t* test (*P<0.05*)) are denoted with *.

As ferritin is the iron storage protein and its expression largely mirrors that of iron levels within the liver, we next assessed the level of ferritin protein in livers. Levels of ferritin protein were determined by Western blotting using a ferritin-specific antibody raised against the heavy chain subunit (Figure 6). Comparison of the band intensity of actin and ferritin found, showed that ferritin protein expression was significantly lower in the rutin-treated group as compared with the control mice (Figure 6B). This is in agreement with reduced iron loading in the livers as seen in the Perls’ staining and the trend seen in the HIC analysis.

## Discussion

Rutin, a flavonol, has both antioxidant and iron chelating properties [15,18,19,30,31]. Rutin treatment has been found to reduce iron levels in animals which were injected with iron to induce iron overload [18,31]. Rutin is able to chelate iron even when given orally [17–20]. This represents a clear advantage over currently used therapeutic iron chelators such as DFO which require prolonged injection times, severely impacting patient compliance rates. However, no studies to date have been conducted on the effect of this compound on animals with a genetic predisposition for iron overload as would be seen in patients with HH. This has resulted in a gap in knowledge about the application of rutin as a therapeutic for disease, as iron overload within humans is more likely to occur due to a genetic mutation that results in dysregulated iron homoeostasis. In the present study, mice which mimic the iron loading pattern of Type III HH were given daily oral treatments of rutin for 21 continuous days.

*Tfr2* KO mice have increased hepatic iron levels after 3 weeks on a standard laboratory chow [32]. Rutin treatment of these mice for 3 weeks resulted in a trend towards reduced HIC and a significant decrease in ferritin expression as would be expected from the removal of iron from this tissue. While ferritin expression can also be influenced by inflammatory stimuli, the levels of neutrophils, monocytes and basophils were not significantly different in the rutin-treated group. This suggests that any effect of inflammation is unlikely to have influenced the change in ferritin expression. The reduction in HIC and ferritin also agree with previous *in vivo* studies which reported reduced hepatic iron after rutin treatment in iron-loaded rats. The pattern of UIBC, TIBC, total iron and TS changes in response to rutin treatment are also in agreement with previously reported data [18]. *Tfr2* KO mice are known to have increased TS as compared with WT mice after 3 weeks of age. Rutin treatment resulted in a significant decrease in TS within these mice. A similar observation of decreased TS was made in iron-loaded rats with rutin treatment [18]. These animals also displayed reduced total iron and TIBC with increased UIBC after rutin treatment. Similar trends for the reduced total iron and UIBC were also seen in the *Tfr2* KO mice treated with rutin. Perls’ staining for hepatic ferric iron also displayed reduced iron deposits in rutin-treated mice compared with the vehicle control. Given the minimal iron levels in spleen and duodenum, rutin treatment did not appear have a substantial effect as shown by Perls’ staining of these tissues. The significant decreases in ferritin protein expression and TS, the trends towards decreased iron content in the liver and serum and the differences observed in iron loading pattern by Perls’ staining taken together indicate that rutin does have the ability to reduce liver and serum iron in this genetic iron overload model.

In recent years, the antioxidant properties of rutin have been investigated for the treatment of serval disorders including, as a neuroprotectant [33], antitumor agent [34] and to prevent hepatotoxicity in alcohol-induced liver injury [35]. These known antioxidant and anti-inflammatory properties of rutin are likely to assist in combating reactive oxygen species (ROS) generation resultant from iron overload. This multimechanism activity of rutin is a key advantage of flavonoids over other chemical compounds for use as a therapeutic.

One explanation for the lack of significant reduction in hepatic iron within the rutin-treated group maybe a result of the different patterns of iron overload. The previous animal model loaded rats with iron via IP injections prior to rutin administration [17,18]. Firstly, this would lead to a different pattern of iron overload as compared with the current study as different cell types would be affected [36]. In addition, daily iron accumulation within the body is approximately equivalent to the loss of iron through mucosal membrane sloughing [37]. The amount of additional iron loading during the rutin-treatment period in the previous studies using iron-injected rats would be minimal. This contrasts with the continual absorption of dietary iron seen in *Tfr2* KO mice where iron loading is seen both before and during the rutin treatment. As the present study only comprised six mice per treatment group, increasing the number of experimental mice and hence the power of the study, would likely result in more statistically significant results.

No significant changes were seen in the gene expression of iron homoeostasis genes in the liver. The reduction in liver iron caused by rutin may have been insufficient to affect the expression levels of some genes, such as *Tfr1*, whose expression levels are usually inversely correlated with cellular iron stores. Under iron overload conditions in wild type animals, *Hamp* expression levels would be expected to decrease upon iron chelation [38] to promote absorption and redistribution of iron stores. However, the absence of a change in *Hamp* in our study is likely due to dysregulated hepcidin regulation resulting from the lack of *Tfr2* in these mice. As TFR2 is known to be involved in iron sensing through the BMP-SMAD pathway, this may also be contributing to the similarity in gene expression between treated and untreated mice of other genes involved in this pathway, such as *Bmp6, Id1* and *Smad7.*

In summary, daily oral rutin treatment of *Tfr2* KO mice for 21 days resulted in significant decreases in liver ferritin protein levels and TS. Taken together with a trend towards decreased hepatic iron content, total serum iron, unsaturated iron binding capacity and a reduction in Perls’ staining in the liver, our results provide evidence that oral rutin treatment does reduce iron stores. The present study provides the first analysis of the iron chelating properties of rutin in a genetic mouse model of HH and the efficacy of rutin for the potential treatment of genetic iron overload disorders such as HH. Future studies will be aimed at determining the effect of increased rutin concentrations and longer treatment time as well as the effect on other genetic models of iron overload and thalassaemia.

## Data Availability

The datasets generated during and/or analysed during the current study are available from the corresponding author on reasonable request.

## Open Access

Open access for this article was enabled by the participation of Queensland University of Technology in an all-inclusive *Read & Publish* pilot with Portland Press and the Biochemical Society under a transformative agreement with CAUL.

## Abbreviations

BMP6: bone morphogenetic protein 6
DIC: duodenal iron concentration
FtnH: ferritin heavy chain
HH: hereditary haemochromatosis
HIC: hepatic iron concentration
Id1: inhibitor of DNA binding 1
KO: knockout
qPCR: real-time quantitative PCR
RBC: red blood cell
ROS: reactive oxygen species
SIC: splenic iron concentration
SMAD: SMA and Mothers Against Decapentaplegic
TBST: Tris buffered saline with 0.1% Tween-20
TF: transferrin
TFR1: transferrin receptor 1
TFR2: transferrin receptor 2
TIBC: total iron binding capacity
TRI: Translational Research Institute
TS: transferrin saturation
UIBC: unsaturated iron binding capacity
UQBRF: University of Queensland Biological Research Facility
